# Preoperative CT-derived liver fat fraction and visceral adipose tissue index enhance postoperative recurrence risk stratification in colorectal cancer

**DOI:** 10.3389/fonc.2026.1944392

**Published:** 2026-09-15

**Authors:** Ke Yin, Mei Wei, Wenjuan Ba, Rongyu Zhou, Bo Xiao

**Affiliations:** 1 Department of Radiology, Bishan Hospital of Chongqing Medical University, Chongqing, China; 2 Department of Radiology, Institute of Tuberculosis Prevention and Treatment of Chongqing, Chongqing, China; 3 Department of Radiology, Chongqing University Affiliated Shapingba Hospital, Chongqing, China; 4 Department of Radiology, Chongqing Tongliang District People’s Hospital, Chongqing, China

**Keywords:** colorectal cancer, computed tomography, liver fat fraction, recurrence-free survival, nomogram, postoperative recurrence, visceral adipose tissue

## Abstract

**Background:**

Postoperative recurrence remains a major cause of treatment failure in colorectal cancer (CRC). We evaluated whether preoperative computed tomography (CT)-derived liver fat fraction (LFF) and visceral adipose tissue index (VATI) improve postoperative recurrence-risk prediction.

**Methods:**

This multi-cohort study included 525 patients after curative-intent surgery: a training cohort (n=260), external validation cohort (n=208), and exploratory prospective cohort (n=57). Candidate clinicopathologic and CT-derived variables were evaluated using Cox regression. Performance was assessed with Harrell C-index, time-dependent area under the receiver operating characteristic curve (AUC) at 12 and 24 months, calibration, decision curve analysis, and Kaplan–Meier analysis.

**Results:**

RFS events were observed in 129 patients. The final model included five predictors. N1–2 stage (hazard ratio [HR], 3.332; 95% confidence interval [CI], 1.807-6.145), elevated carbohydrate antigen 19-9 (CA19-9; HR, 2.414; 95% CI, 1.459-3.994), lymphovascular invasion (HR, 2.166; 95% CI, 1.250-3.752), VATI per 1-standard-deviation increase (HR, 1.519; 95% CI, 1.217-1.896), and LFF per 1-standard-deviation increase (HR, 2.228; 95% CI, 1.728-2.871) were independently associated with recurrence-free survival. C-indices were 0.851, 0.809, and 0.828 in the training, external, and prospective cohorts, respectively. Prospective 12- and 24-month AUCs were 0.759 (95% CI, 0.242-1.000) and 0.890 (95% CI, 0.709-1.000), with the latter based on only 20 evaluable patients. The combined validation cohort yielded a C-index of 0.813 and 12- and 24-month AUCs of 0.807 and 0.860.

**Conclusion:**

CT-derived VATI and LFF improved risk stratification beyond routine clinicopathologic variables. The prospective findings remain exploratory and require confirmation in larger cohorts before clinical implementation.

## Introduction

Colorectal cancer (CRC) remains a major global health burden. Despite advances in screening, surgery, systemic therapy, and surveillance, distant recurrence after curative-intent surgery remains a major determinant of long-term outcome. Recent nationwide data confirm clinically meaningful recurrence after stage I–III CRC, particularly during the early postoperative years (1–3). More precise risk stratification may identify patients needing closer surveillance or individualized management. Current prognostic assessment relies mainly on pathological stage, nodal status, LVI, perineural invasion, histologic grade, and tumor markers (4, 5). These factors do not fully explain heterogeneous postoperative recurrence risk. Different outcomes among patients with similar pathological stage suggest that host metabolic characteristics may provide additional prognostic information.

Preoperative computed tomography (CT) provides host metabolic information without additional imaging. CT body-composition analysis measures visceral, subcutaneous, and intermuscular adipose tissue at standardized anatomical levels. CT-derived body-composition measures have been associated with CRC outcomes (6–8). Similar findings were reported in early-stage and rectal cancer (9, 10). Systematic reviews show heterogeneous effects (11, 12). Other recent cohorts confirm variation by disease stage and measurement strategy (13, 14). Hepatic fat is another metabolic feature available from abdominal imaging and may be relevant in CRC because the liver is a frequent site of distant metastasis. Steatosis may alter lipid metabolism, oxidative stress, immune signaling, and the hepatic inflammatory microenvironment. Hepatic steatosis has been linked to CRC recurrence and liver metastasis (15–17). Steatotic liver disease is also associated with extrahepatic cancer risk (18, 19). Visceral adipose tissue is metabolically active and has been linked to insulin resistance, systemic inflammation, adipokine imbalance, and immune modulation (20–22). Few prognostic models have integrated CT-derived liver fat fraction (LFF) and visceral adiposity.

We investigated whether preoperative CT-derived LFF and visceral adipose tissue index (VATI) were associated with postoperative recurrence in CRC. We then developed and validated a Cox-based nomogram combining significant clinicopathologic and CT-derived predictors across internal training, external validation, and internal prospective validation cohorts.

## Materials and methods

### Study population

The study was conducted in accordance with the Declaration of Helsinki and approved by the Institutional Ethics Review Committee of Bishan Hospital of Chongqing Medical University (cqbyklyy-20240918-24). Consent was waived for the anonymized retrospective component; written informed consent was obtained for prospective enrollment. Retrospective patients were treated from January 2019 to January 2023 at Bishan Hospital of Chongqing Medical University (primary center) and two external centers (Chongqing University Affiliated Shapingba Hospital and Chongqing Tongliang District People’s Hospital). The prospective cohort was enrolled consecutively at the primary center from June 2024 to January 2025.

The multi-cohort design was center- and time-based rather than randomly split. Eligible patients had pathologically confirmed T2–T3 CRC, curative-intent surgical resection, preoperative abdominal CT suitable for VATI/LFF quantification, and complete key clinicopathologic and follow-up data. Patients with pathological T1 disease were not included because all such patients at the participating centers were managed by endoscopic resection rather than conventional surgical resection. Exclusion criteria were synchronous distant metastasis, incomplete adjuvant chemotherapy, another primary malignancy, neoadjuvant treatment or non-primary diagnosis at the participating center, loss to follow-up, or CT unsuitable for quantitative analysis. Primary-center retrospective cases formed the training cohort. Retrospective cases from the two external hospitals formed an independent geographic validation cohort. The later primary-center prospective cohort provided exploratory temporal validation.

Among 710 retrospectively assessed patients, 242 were excluded (12 incomplete adjuvant chemotherapy, 16 other primary malignancy, 21 neoadjuvant treatment/non-primary diagnosis, 79 lost to follow-up, and 114 metastatic CRC at diagnosis), leaving 468 patients: 260 for training and 208 for external validation. Among 85 prospectively assessed patients, 28 were excluded (1 incomplete adjuvant chemotherapy, 3 neoadjuvant treatment/non-primary diagnosis, 9 lost to follow-up, and 15 metastatic CRC at diagnosis), leaving 57 patients. Overall, 525 patients were analyzed (**Figure 1**). The prospective cohort was evaluated using the same definitions, training-cohort standardization parameters, fixed Cox coefficients, and training-derived risk threshold, without model refitting.

**Figure 1:**
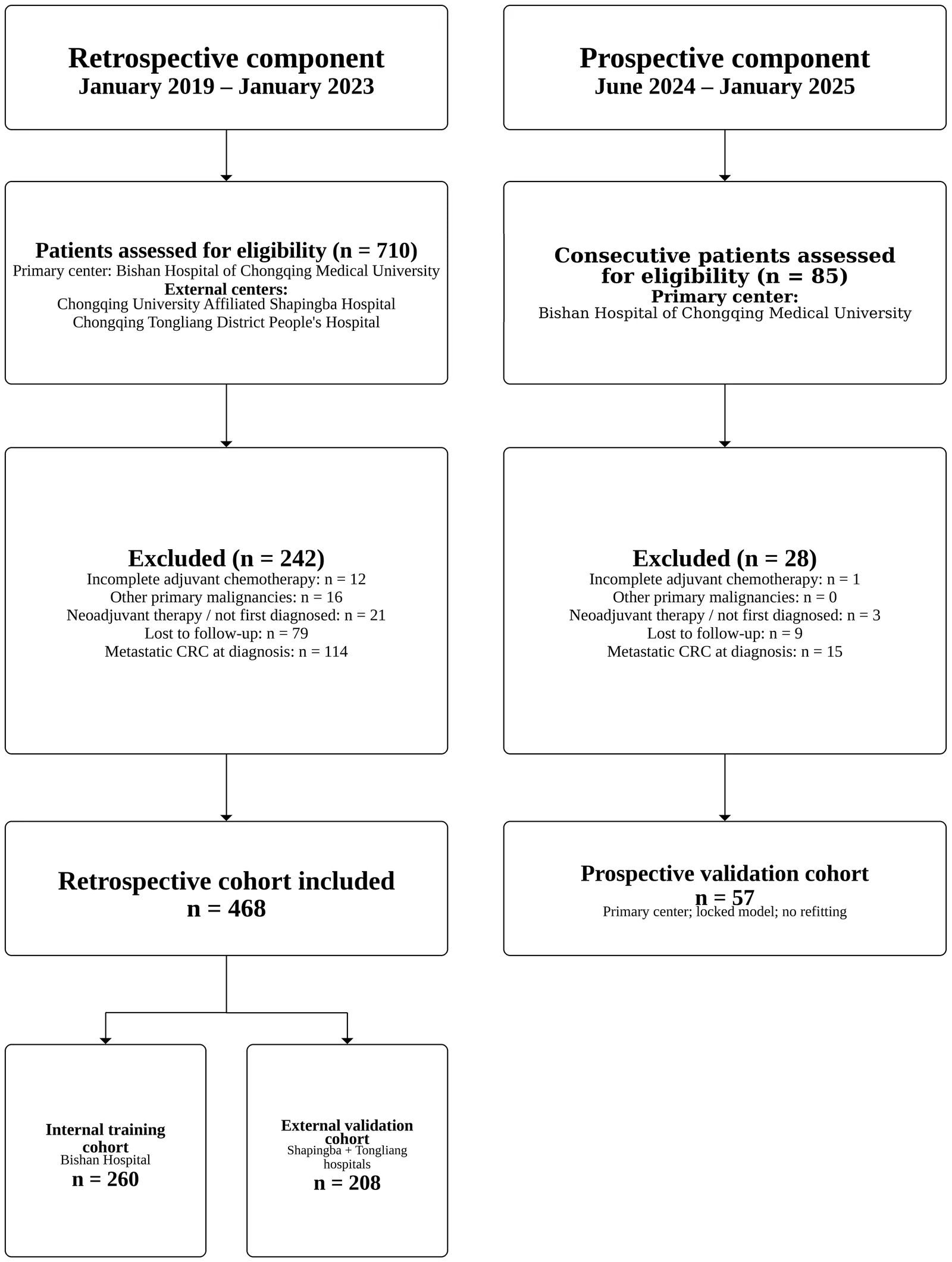
Flow diagram of patient enrollment and cohort allocation. The retrospective component covered January 2019 to January 2023. Of 710 patients assessed across the primary center and two external hospitals, 242 were excluded, leaving 260 patients for internal training and 208 for external validation. The prospective component enrolled patients at the primary center from June 2024 to January 2025; 85 were assessed, 28 were excluded, and 57 formed the exploratory prospective validation cohort. Cohort allocation was center- and time-based rather than random. CRC, colorectal cancer.

Postoperative surveillance consisted of carcinoembryonic antigen (CEA), carbohydrate antigen 19-9 (CA19-9), and chest/abdominal/pelvic CT every 3 months during years 0–2, every 6 months during year 3, and annually during years 4–5. Positron emission tomography/CT (PET/CT) was reserved for suspected recurrence based on CT and/or tumor-marker findings. Colonoscopy was scheduled at years 1 and 3.

### Clinical and pathologic variables

Clinical variables included sex, age, T and N stage, preoperative CEA and CA19-9, PNI, LVI, histologic grade, body mass index category, and tumor location. N stage was categorized as N0 versus N1–2; tumor markers used institutional reference limits; tumor location was colon or rectum.

### CT-derived VATI and LFF assessment

Preoperative unenhanced abdominal CT was acquired on a GE Revolution CT, GE Discovery CT750 HD, or Siemens SOMATOM Definition AS using 120 kVp, automatic tube-current modulation, and a 512 × 512 matrix. Original images were reconstructed at 5-mm thickness and 5-mm interval; an additional 1-mm reconstruction using a standard soft-tissue kernel was used for quantitative analysis. Body composition was measured on a prespecified single axial slice through the mid-L3 vertebral body with semi-automated Bones QCT software (Bones Technology Limited, Hong Kong, China), followed by reader review and manual boundary correction when needed. Visceral and intermuscular adipose tissue were segmented at −150 to −30 HU and subcutaneous adipose tissue at −190 to −50 HU. VATI, IMATI, and SATI were calculated as tissue area (cm²)/height² (m²), and VSR as visceral/subcutaneous fat area. L3 was selected *a priori* as a standardized practical surrogate for whole-body adiposity (23–25). Two trained readers independently reviewed segmentations (26).

For LFF assessment, four circular ROIs were placed on unenhanced CT in the left lateral, left medial, right anterior, and right posterior liver, avoiding visible large vessels and focal lesions where possible. Mean attenuation was CT-HUmean = (HUleft lateral + HUleft medial + HUright anterior + HUright posterior)/4, and CT-LFF (%) = −0.58 × CT-HUmean + 38.2 (27). This equation was derived from paired unenhanced 120-kV CT and magnetic resonance imaging proton-density fat fraction (MRI-PDFF); the primary calibration cohort showed R²=0.828 when CT and MRI were obtained within 1 month (27) (**Figure 2**). Images had to permit reliable mid-L3 segmentation and four-region liver attenuation measurement. Severe artifact or anatomic distortion precluding quantification led to exclusion; correctable segmentation errors were manually adjusted.

**Figure 2:**
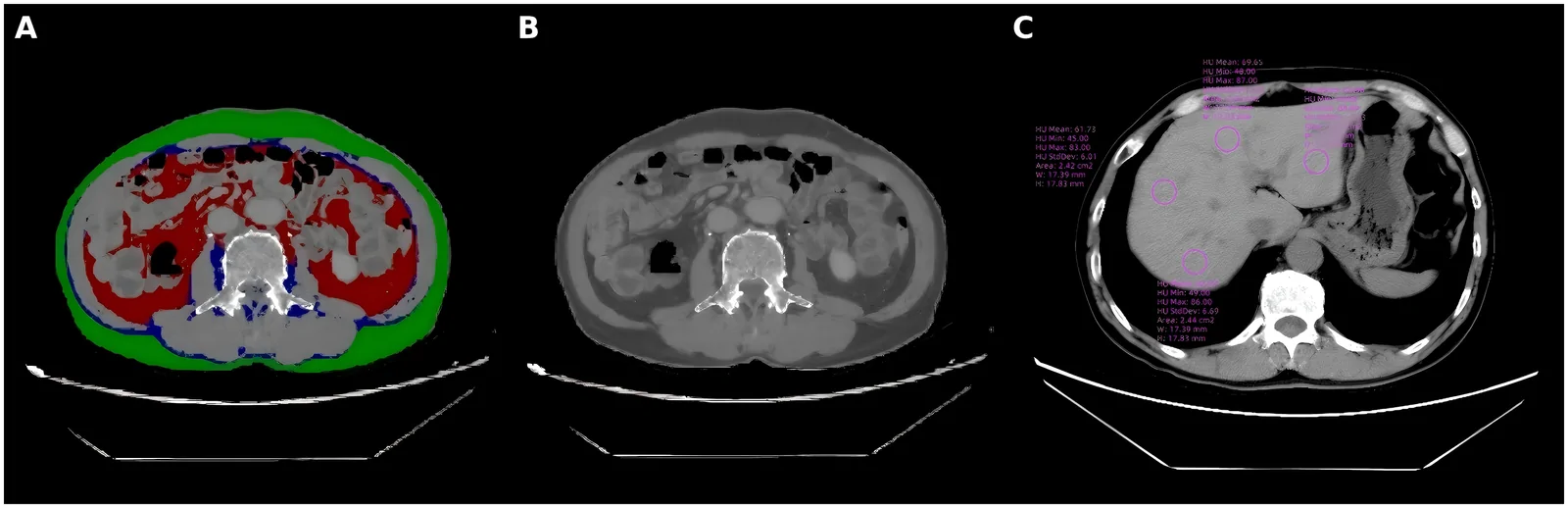
Representative computed tomography (CT) assessment. **(A)** Semi-automated mid-L3 body-composition segmentation. Visceral and intermuscular adipose tissue were segmented from −150 to −30 Hounsfield units (HU), and subcutaneous adipose tissue from −190 to −50 HU. **(B)** Corresponding unenhanced axial CT image. **(C)** Four hepatic regions of interest used to calculate mean liver attenuation and CT-derived liver fat fraction (CT-LFF): CT-HUmean = (HUleft lateral + HUleft medial + HUright anterior + HUright posterior)/4; CT-LFF (%) = −0.58 × CT-HUmean + 38.2.

For model development, continuous variables, including age, IMATI, SATI, VATI, VSR, and LFF, were standardized using the mean and standard deviation (SD) of the internal training cohort. Training-cohort SDs were 12.402 cm²/m² for VATI and 4.440 percentage points for LFF. Standardized hazard ratios (HRs) were retained for comparison across biomarkers; the same Cox models were also expressed on the original scales as HRs per 1 cm²/m² increase in VATI and per 1-percentage-point increase in LFF. Training-derived standardization parameters were applied unchanged to all validation cohorts.

### Outcome

The primary endpoint was recurrence-free survival (RFS), defined as the interval from curative-intent surgery to the first documented distant metastasis or local recurrence at the operative site/anastomosis. Events were identified on follow-up cross-sectional imaging and clinical assessment, with PET/CT and/or histopathologic confirmation when clinically obtained. Patients without either event were censored at the last documented event-free assessment. The nomogram estimated 12- and 24-month RFS.

### Statistical analysis

Continuous variables were summarized as medians with interquartile ranges, and categorical variables as numbers with percentages. Cohort differences were assessed using nonparametric tests for continuous variables and chi-square or Fisher exact tests for categorical variables, as appropriate. Measurement reproducibility was evaluated using single-measure absolute-agreement intraclass correlation coefficients (ICCs). VATI interobserver agreement was assessed in a 50-examination subset; LFF interobserver and intraobserver agreement were assessed in all 525 examinations. Bland–Altman analysis, Wilcoxon signed-rank tests, Pearson correlation, and bootstrap 95% confidence intervals were additionally used (**Supplementary Table S1**; **Supplementary Figure S1**).

Model development was performed in the training cohort. Variables with P <.05 in univariable Cox regression entered multivariable analysis, and independently significant predictors formed the nomogram. Multicollinearity was assessed using variance inflation factors (VIFs), and the proportional-hazards assumption using Schoenfeld residuals. Robustness was examined using 10-fold cross-validated least absolute shrinkage and selection operator (LASSO) Cox regression and 1, 000-resample bootstrap validation (28–30). Events per variable (EPV), a missing-data audit, within-cohort comparisons, and prespecified interaction analyses were also performed (31, 32). Benjamini–Hochberg adjustment was applied to the eight imaging-biomarker interaction tests (**Supplementary Tables S2**, **S3**).

Model discrimination used Harrell C-index and time-dependent AUCs (33, 34). Calibration and decision curve analysis were assessed at 12 and 24 months (35–37). Candidate models were compared across cohorts, and continuous predicted probabilities were the primary outputs. For exploratory risk stratification, the training-cohort median linear predictor defined fixed low- and high-risk groups and was applied unchanged to the validation cohorts and tumor-location and pathological-stage subgroups; Kaplan–Meier curves were compared using log-rank tests. Alternative risk thresholds and time-specific VATI/LFF cutoffs were examined in sensitivity analyses (**Supplementary Table S4**; **Supplementary Figure S2**).

The external and prospective cohorts were evaluated separately and pooled for combined validation. Prospective C-index confidence intervals were estimated using 10, 000 bootstrap resamples, and time-specific AUC confidence intervals were reported. A *post hoc* precision-based sample-size assessment for time-to-event model validation followed Riley et al. (38), using the combined validation cohort as the empirical target population and prespecified confidence-interval-width criteria. All tests were two-sided, and P <.05 indicated statistical significance.

## Results

### Patient characteristics

A total of 525 patients were included: 260 in training, 208 in external validation, and 57 in prospective validation. RFS events occurred in 64, 52, and 13 patients, respectively (129 overall). Median age was 66 years (IQR, 57-73), and median follow-up was 21 months (IQR, 13-29). Baseline characteristics were generally comparable across cohorts (**Table 1**). N stage, CA19-9, LVI, VATI, LFF, and tumor location did not differ significantly (all P >.46). Within-cohort event-status comparisons are summarized in **Supplementary Table S3**; RFS events were generally associated with adverse pathologic or tumor-marker features and higher LFF, while VATI differed only in the training cohort.

**Table 1:** Baseline characteristics across the three cohorts.

Variable	Overall (n=525)	Training (n=260)	External (n=208)	Prospective (n=57)	P value
Age, years	66 [57-73]	68 [57-74]	66 [58-73]	62 [55-71]	0.157
Male sex	271 (51.6)	124 (47.7)	111 (53.4)	36 (63.2)	0.086
T3 stage	447 (85.1)	228 (87.7)	175 (84.1)	44 (77.2)	0.114
N1–2 stage	233 (44.4)	119 (45.8)	93 (44.7)	21 (36.8)	0.467
Elevated CEA	207 (39.4)	102 (39.2)	90 (43.3)	15 (26.3)	0.067
Elevated CA19-9	123 (23.4)	60 (23.1)	48 (23.1)	15 (26.3)	0.862
PNI positive	230 (43.8)	105 (40.4)	99 (47.6)	26 (45.6)	0.283
LVI positive	222 (42.3)	111 (42.7)	87 (41.8)	24 (42.1)	0.982
VATI, cm²/m²	38.27 [32.01-47.06]	37.64 [31.63-45.31]	40.51 [32.04-47.10]	36.40 [30.85-48.07]	0.556
LFF, %	2.82 [0.21-6.01]	2.53 [-0.08-6.01]	2.82 [1.01-5.79]	2.82 [0.50-6.01]	0.513
Rectal cancer	167 (31.8)	83 (31.9)	63 (30.3)	21 (36.8)	0.641
Follow-up, mo	21 [13-29]	22 [12.75-30]	22 [12.75-32]	19 [17-23]	0.239
RFS event	129 (24.6)	64 (24.6)	52 (25.0)	13 (22.8)	0.943

Values are median [interquartile range] or n (%). P values compare the three cohorts. CA19-9, carbohydrate antigen 19-9; CEA, carcinoembryonic antigen; LFF, liver fat fraction; LVI, lymphovascular invasion; RFS, recurrence-free survival; PNI, perineural invasion; VATI, visceral adipose tissue index.

### Imaging measurement reproducibility

VATI interobserver reproducibility was excellent (ICC, 0.995; 95% CI, 0.991-0.997). LFF interobserver and intraobserver ICCs were 0.856 (95% CI, 0.822-0.883) and 0.896 (95% CI, 0.870-0.917), respectively. Mean paired differences were small and nonsignificant; Bland–Altman limits and correlations are reported in **Supplementary Table S1**, **Supplementary Figure S1**.

### Cox regression and final model

Univariable Cox analysis identified T stage, N stage, CEA, CA19-9, PNI, LVI, VATI, LFF, and tumor location as associated with RFS (**Table 2**). In multivariable analysis, N stage, CA19-9, LVI, VATI, and LFF remained significant and formed the final nomogram (**Figure 3**); T stage, CEA, PNI, and tumor location were not retained (**Supplementary Table S2**). N1–2 stage (HR, 3.332; 95% CI, 1.807-6.145) and elevated CA19-9 (HR, 2.414; 95% CI, 1.459-3.994) (both P <.001) predicted poorer RFS, as did LVI (HR, 2.166; 95% CI, 1.250-3.752; P = .006). Each 1-SD increase in VATI (HR, 1.519; 95% CI, 1.217-1.896) and LFF (HR, 2.228; 95% CI, 1.728-2.871) was also adverse (both P <.001); native-scale estimates are shown in **Table 2**.

**Table 2:** Cox regression analysis in the internal training cohort.

Variable	Univariable HR (95% CI)	P value	Final model HR (95% CI)	P value
LFF (per 1 SD)	2.022 (1.611-2.538)	<.001	2.228 (1.728-2.871)	<.001
LFF (per 1 percentage point)	1.172 (1.114-1.234)	<.001	1.198 (1.131-1.269)	<.001
N1–2 vs N0	4.630 (2.595-8.260)	<.001	3.332 (1.807-6.145)	<.001
Elevated CA19-9	3.294 (2.010-5.399)	<.001	2.414 (1.459-3.994)	<.001
LVI positive	3.501 (2.070-5.919)	<.001	2.166 (1.250-3.752)	.006
Elevated CEA	2.858 (1.726-4.730)	<.001	—	—
PNI positive	2.658 (1.608-4.391)	<.001	—	—
VATI (per 1 SD)	1.464 (1.190-1.800)	<.001	1.519 (1.217-1.896)	<.001
VATI (per 1 cm²/m²)	1.031 (1.014-1.049)	<.001	1.034 (1.016-1.053)	<.001
Rectum vs colon	2.263 (1.384-3.700)	.001	—	—
T3 vs T2	4.495 (1.099-18.383)	.036	—	—

Only variables significant in univariable Cox regression are shown. Final-model estimates are reported for the five predictors retained in the nomogram. For VATI and LFF, both standardized (per 1 SD) and original-unit HRs are shown; the latter were obtained by expressing the same Cox coefficients on the native scales (VATI per 1 cm²/m² and LFF per 1 percentage point). Adjusted estimates for all nine variables in the candidate multivariable model, together with VIF diagnostics, CEA-by-CA19-9/T-by-N interaction analyses, cross-validated LASSO Cox analysis, and bootstrap internal-validation/shrinkage results are provided in **Supplementary Table S2**. Exploratory time-specific VATI/LFF cutoffs are provided in **Supplementary Table S4**. CA19-9, carbohydrate antigen 19-9; CEA, carcinoembryonic antigen; CI, confidence interval; HR, hazard ratio; LASSO, least absolute shrinkage and selection operator; LFF, liver fat fraction; LVI, lymphovascular invasion; PNI, perineural invasion; SD, standard deviation; VATI, visceral adipose tissue index; VIF, variance inflation factor.

**Figure 3:**
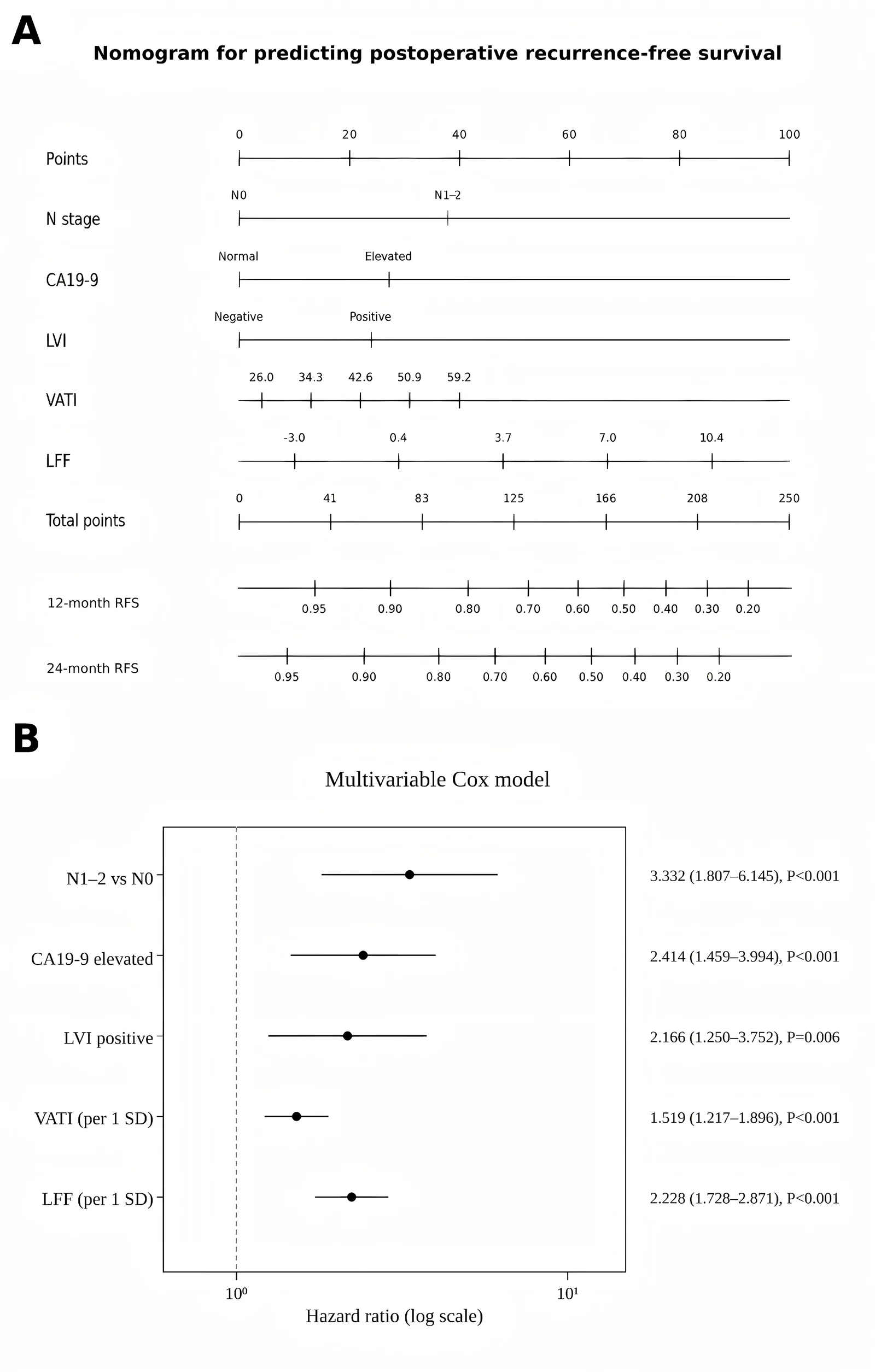
Final nomogram and multivariable Cox model. **(A)** Nomogram for predicting 12- and 24-month recurrence-free survival (RFS) using N stage, carbohydrate antigen 19-9 (CA19-9), lymphovascular invasion (LVI), visceral adipose tissue index (VATI), and liver fat fraction (LFF). **(B)** Forest plot of the final Cox model. VATI and LFF are shown per 1-standard-deviation increase. Hazard ratios and 95% confidence intervals are identical to the final-model estimates in **Table 2**.

No proportional-hazards violations were detected for the final predictors or globally (global P = .994; **Supplementary Table S2**). Robustness analyses showed no problematic collinearity or marked overfitting. Cross-validated LASSO retained all five final predictors; bootstrap correction changed the C-index from 0.851 to 0.839 (optimism, 0.012) and yielded a calibration slope of 0.927 (**Supplementary Table S2**). The CEA-by-CA19–9 interaction was not significant.

Time-specific VATI and LFF cutoffs varied across horizons and transported inconsistently to external validation, with stronger discrimination for LFF than VATI (**Supplementary Table S4**). EPVs were 7.1 for the nine-variable candidate model and 12.8 for the final model. None of eight exploratory biomarker-by-clinical-factor interactions remained significant after multiplicity correction (minimum q = .126; **Supplementary Table S2**).

### Nomogram performance and model comparison

Final-model C-indices were 0.851 in training, 0.809 in external validation, 0.828 in prospective validation, and 0.813 in combined validation (**Table 3**). In the prospective cohort, the C-index was 0.828 (95% CI, 0.675-0.947), the 12-month AUC was 0.759 (95% CI, 0.242-1.000; 57 evaluable patients, 3 cases), and the 24-month AUC was 0.890 (95% CI, 0.709-1.000; 20 evaluable patients, 13 cases). Combined-validation AUCs were 0.807 (95% CI, 0.721-0.893) and 0.860 (95% CI, 0.797-0.915) at 12 and 24 months, respectively (**Figures 4A, B**; **Table 3**). A *post hoc* precision analysis indicated that approximately 580 participants and 142 events would be required for definitive external validation. Calibration and decision curve analyses at 12 and 24 months are presented in **Figure 5**.

**Table 3:** Discrimination performance of the final nomogram at 12 and 24 months.

Cohort	N	Events	C-index	12-month AUC	24-month AUC
Internal training	260	64	0.851	0.918	0.878
External validation	208	52	0.809	0.810	0.859
Prospective validation (exploratory)	57	13	0.828	0.759	0.890
Combined validation	265	65	0.813	0.807	0.860
All patients	525	129	0.833	0.862	0.868

AUC, area under the receiver operating characteristic curve; C-index, concordance index; CI, confidence interval. In the exploratory prospective cohort, the C-index was 0.828 (95% CI, 0.675-0.947), the 12-month AUC was 0.759 (95% CI, 0.242-1.000), and the 24-month AUC was 0.890 (95% CI, 0.709-1.000). The 24-month prospective AUC was based on 20 evaluable patients (13 cases and 7 controls). In the combined validation cohort, the 12- and 24-month AUCs were 0.807 (95% CI, 0.721-0.893) and 0.860 (95% CI, 0.797-0.915), respectively.

**Figure 4:**
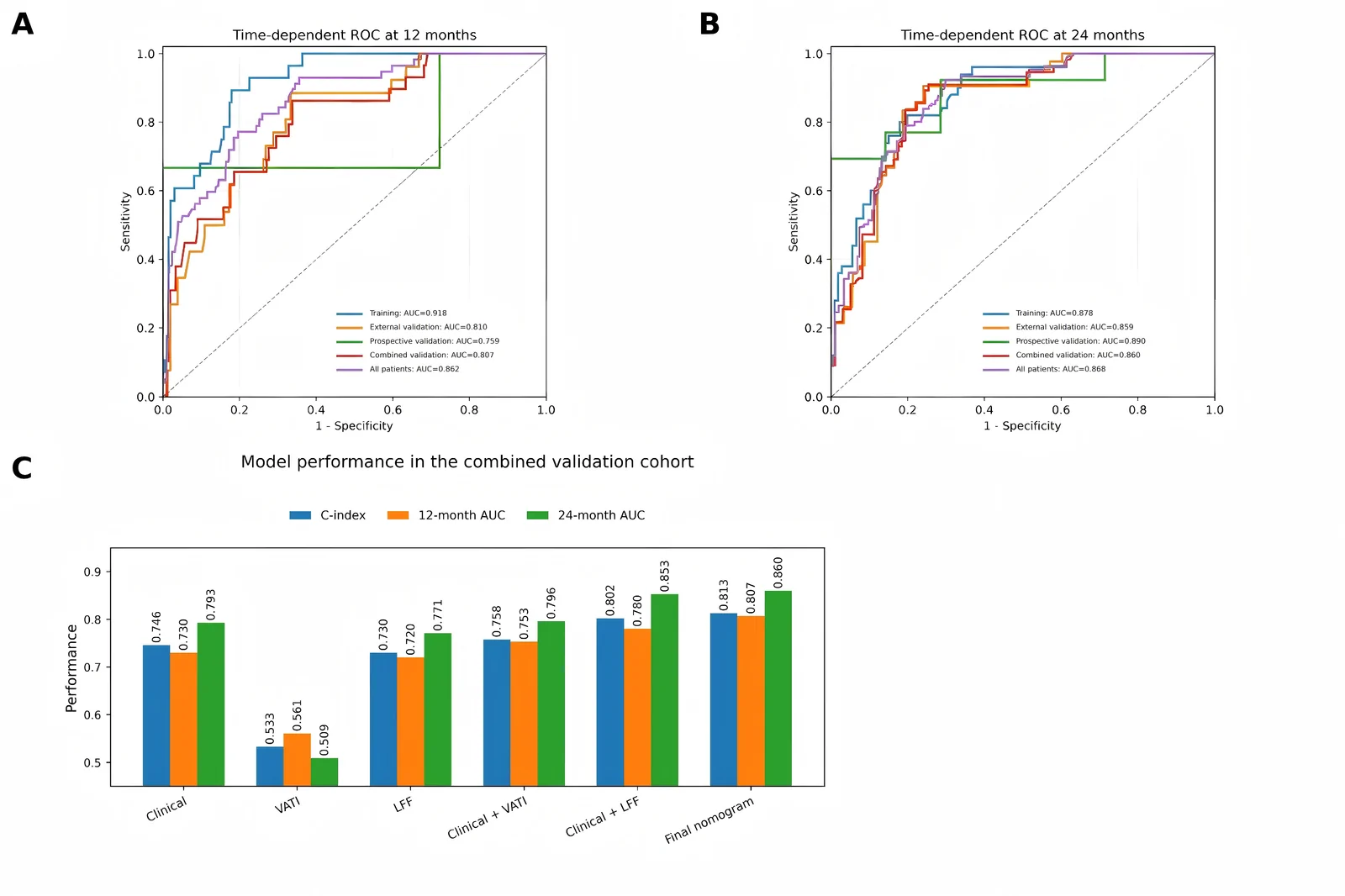
Discrimination performance of the final nomogram. **(A)** Time-dependent receiver operating characteristic (ROC) curve for 12-month recurrence-free survival (RFS). **(B)** Time-dependent ROC curve for 24-month RFS. **(C)** C-index and area under the ROC curve (AUC) across candidate models in the combined validation cohort. Complete model-by-cohort comparisons are provided in **Supplementary Table S5**.

**Figure 5:**
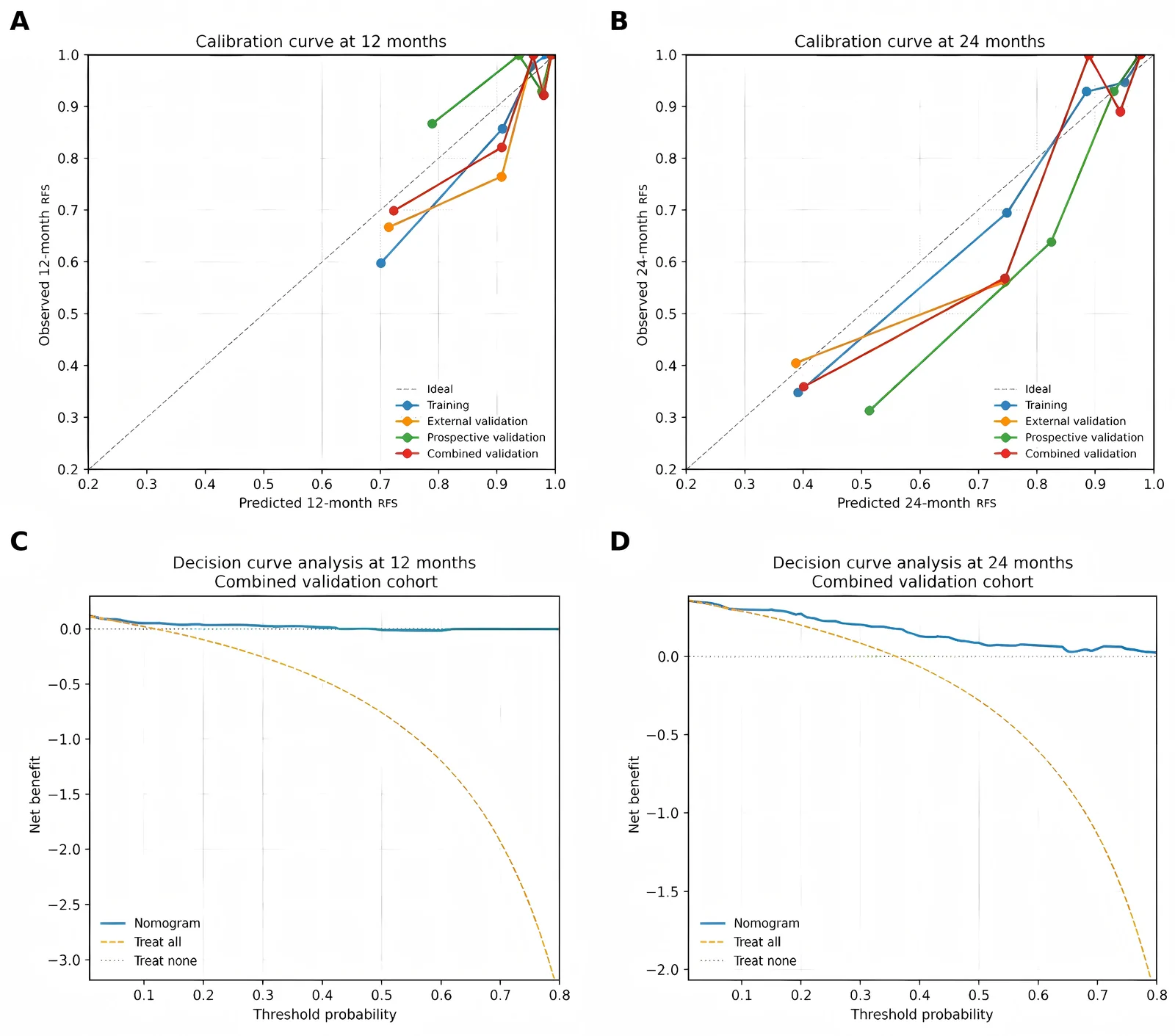
Calibration and decision curve analyses of the final nomogram. **(A)** Calibration at 12 months and **(B)** at 24 months for recurrence-free survival (RFS). **(C)** Decision curve analysis at 12 months and **(D)** at 24 months in the combined validation cohort.

The final nomogram numerically outperformed the clinical-only model in each cohort; C-index increased from 0.776 to 0.851 in training, 0.743 to 0.809 in external validation, and 0.748 to 0.828 prospectively (**Supplementary Table S5**; **Figure 4C**). VATI alone had limited discrimination, LFF was stronger, and their integration produced the most consistent performance.

### Risk stratification

The fixed training-median linear predictor (1.011232) separated RFS in training, external, prospective, colon, and rectal cohorts (**Figure 6**; **Supplementary Table S4**). High-versus-low HRs were 19.89 (95% CI, 7.21-54.86) in training and 12.91 (95% CI, 5.56-29.97) in combined validation (both P <.001). Alternative thresholds also separated risk but produced more imbalanced groups; grouped calibration is shown in **Supplementary Figure S2**.

**Figure 6:**
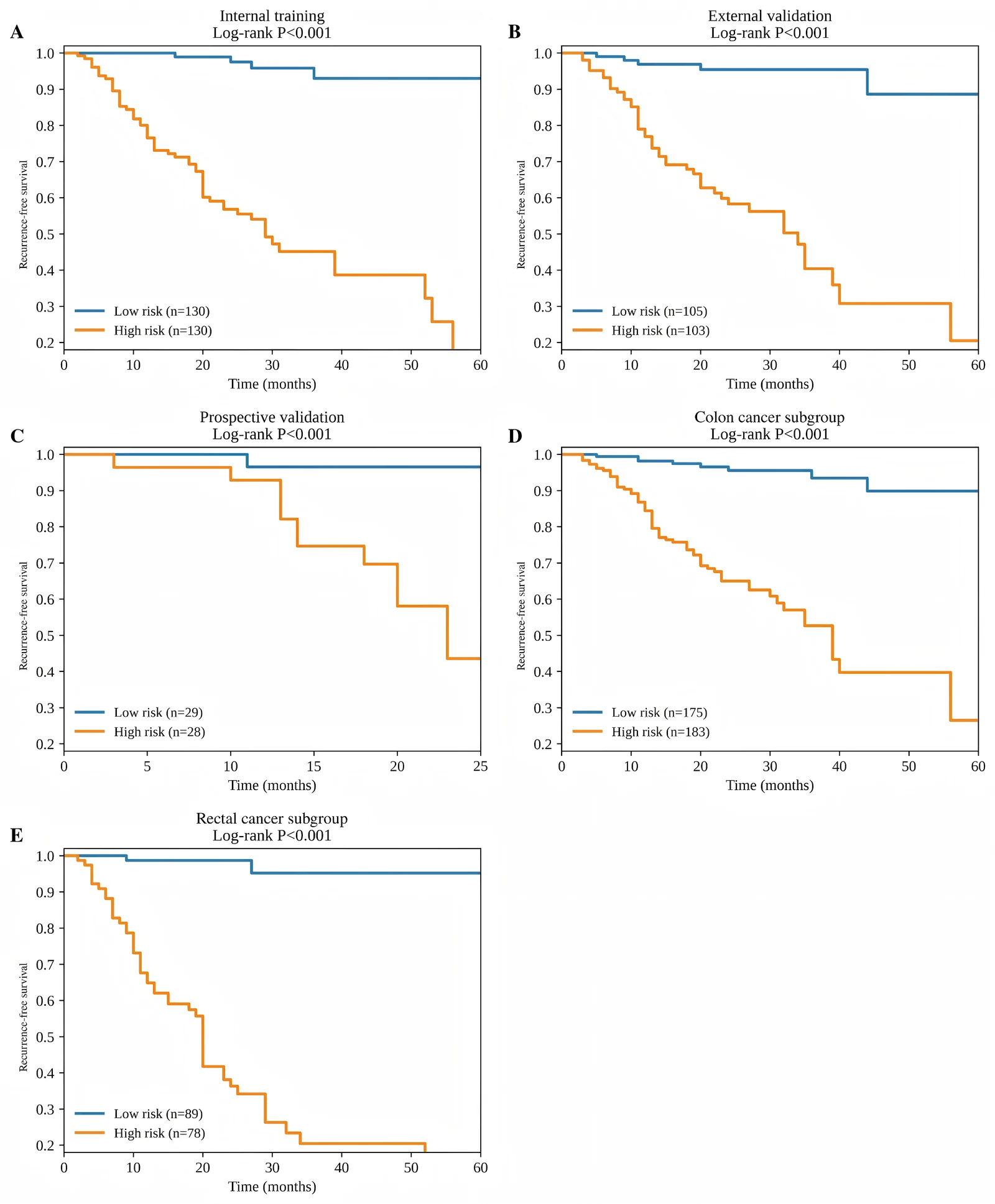
Kaplan–Meier curves for RFS according to model-based risk groups. **(A)** Internal training cohort. **(B)** External validation cohort. **(C)** Internal prospective validation cohort. **(D)** Colon cancer subgroup. **(E)** Rectal cancer subgroup. The training-cohort median linear predictor (1.011232) was applied unchanged across cohorts and subgroups. Comparative cutoff analyses are provided in **Supplementary Table S4**; continuous nomogram probability remains the primary prognostic output.

The same fixed cutoff separated low- and high-risk patients within stages I (65 patients; 4 events), II (227, 33), and III (233, 92) (all log-rank P <.001; **Supplementary Figure S3**).

## Discussion

This study developed a CT-based nomogram integrating N stage, CA19-9, LVI, VATI, and LFF. The model numerically outperformed the clinical-only model in the training, external validation, and exploratory prospective cohorts. VATI alone had limited discrimination, LFF was more informative, and their combination with clinicopathologic factors produced the most consistent performance. Fixed risk groups also separated RFS across pathological stages I–III. N stage, LVI, and CA19–9 retained independent prognostic value, whereas CEA, PNI, T stage, and tumor location did not remain significant after multivariable adjustment.

Beyond the imaging biomarkers, the independent contributions of N stage, preoperative CA19-9, and LVI support the clinicopathological plausibility of the model. Among the conventional variables, N1–2 stage showed the strongest association with poorer RFS. Nodal involvement reflects established regional dissemination, whereas LVI provides direct histopathological evidence of tumor access to lymphatic or vascular channels; both are recognized markers of postoperative recurrence risk and inform adjuvant management in localized CRC (4, 5). Elevated preoperative CA19–9 may capture additional variation in occult tumor burden or aggressive tumor biology and has shown prognostic value when combined with CT-derived body-composition measures (39). Their integration with VATI and LFF therefore combines tumor extent and invasive behavior with host metabolic phenotypes, which may explain the more consistent performance of the final nomogram than models based on either clinical or imaging variables alone.

Higher VATI was independently associated with poorer RFS. Visceral adipose tissue may promote insulin resistance, free-fatty-acid flux, inflammatory adipokine signaling, and immune-metabolic dysregulation, while portal drainage could connect these systemic effects to the hepatic microenvironment (20–22). Experimental evidence also links obesity-related fatty-acid availability to DGAT1/2-dependent lipid-droplet remodeling in CRC (40). However, VATI showed weak standalone discrimination and horizon-dependent cutoffs. It is therefore better interpreted as a continuous host-risk phenotype than as a binary test.

LFF showed a stronger standardized association with RFS than VATI. Hepatic steatosis may alter lipid flux, oxidative stress, and stromal and endothelial remodeling (15–17). These changes may impair immune surveillance and facilitate metastatic colonization (18, 19). Mechanistic work further links steatosis to poor-prognosis replacement-pattern colorectal liver metastases through fatty-acid oxidation, MYC stabilization, proline synthesis, and collagen production (41). In external validation, LFF-based models performed better than corresponding VATI-based models, while the combined nomogram performed best. VATI may therefore reflect broader host adiposity, whereas LFF may capture a more proximal hepatic metabolic-inflammatory state; this complementary portal-metabolic interpretation remains hypothesis-generating (39, 42).

CT-LFF is an attenuation-derived MRI-PDFF-equivalent estimate rather than a direct measure of hepatic triglyceride content. Although the published 120-kV calibration correlated well with MRI-PDFF (R²=0.828) (27), scanner and reconstruction differences may affect absolute attenuation. Interobserver and intraobserver ICCs were 0.856 and 0.896, but prospective cross-scanner validation against closely timed MRI-PDFF remains necessary. The external cohort provided the principal validation, whereas the prospective cohort offered exploratory temporal support and had wide confidence intervals. Because outcome-optimized cutoffs varied and produced imbalanced groups, continuous predicted probabilities should remain the primary model output.

Clinical translation will require standardized CT acquisition, validated automated or semi-automated measurements, cross-scanner harmonization, and quality control. A practical workflow could combine preoperative VATI, LFF, and CA19–9 with postoperative pathological N stage and LVI to generate continuous 12- and 24-month RFS probabilities for multidisciplinary review. Automated body-composition assessment is feasible in CRC (7), but liver ROI placement and failure detection require further validation. Until clinical-impact studies are available, the nomogram should be used only as an adjunct to conventional risk assessment and should not independently alter adjuvant therapy or surveillance.

This study has several limitations. Its observational design leaves residual confounding, and the prospective cohort was small (57 patients, 13 events), with only 20 patients evaluable for the 24-month AUC. Exclusion of neoadjuvant-treated patients limits applicability to locally advanced rectal cancer. Significance-based selection may be unstable despite supportive VIF, LASSO, and bootstrap analyses. Treatment and molecular variables, including adjuvant regimen, RAS/BRAF, MMR/MSI, and postoperative ctDNA, were unavailable (4, 5, 43). Molecular residual disease was not evaluated (44, 45). Metabolic syndrome, chronic liver disease, and relevant medications were also unavailable. VATI was measured on one L3 slice, and CT-LFF lacked local MRI-PDFF or histologic validation. Loss to follow-up and surveillance variability may have introduced bias, while death without an RFS event was not modeled. Finally, metastasis site and longitudinal changes in adiposity, liver fat, treatment exposure, and inflammation were unavailable.

In conclusion, preoperative CT-derived VATI and LFF were independently associated with RFS after curative-intent CRC surgery. Their integration with N stage, CA19-9, and LVI showed consistent discrimination in training and external validation, with exploratory prospective support. Larger prospective studies and standardized imaging workflows are required before clinical use.

## Data Availability

The original contributions presented in the study are included in the article/**Supplementary Material**. Further inquiries can be directed to the corresponding author.
