# Supplementary material for: Preoperative CT-derived liver fat fraction and visceral adipose tissue index enhance postoperative recurrence risk stratification in colorectal cancer
Source: Front Oncol. 2026 Sep 15;16:1944392. doi: 10.3389/fonc.2026.1944392 (PMC13619508; doi:10.3389/fonc.2026.1944392)
Supplement: Supplementary file 1 [file SupplementaryFile1.docx]

**Supplementary Figures**

**Supplementary Figure S1**

**Supplementary Figure S1.** Bland–Altman analysis of CT-derived liver fat fraction reproducibility. (A) Interobserver agreement between the initial Reader 1 measurement and Reader 2 measurement. (B) Intraobserver agreement between the initial and repeat Reader 1 measurements. Solid horizontal lines indicate the mean measurement difference, and dashed lines indicate the 95% limits of agreement.

**Supplementary Figure S2**

**Supplementary Figure S2.** Grouped calibration of the fixed low- and high-risk categories in the combined validation cohort. (A) Twelve-month calibration. (B) Twenty-four-month calibration. Points show mean predicted RFS-event probability versus Kaplan–Meier observed RFS-event probability for the low- and high-risk groups; vertical error bars indicate 95% confidence intervals for the observed probabilities. The dashed diagonal represents ideal calibration.

**Supplementary Figure S3**

**Supplementary Figure S3.** Kaplan–Meier curves for fixed nomogram risk groups stratified by pathological stage. (A) Stage I (T2N0; n=65, 4 events). (B) Stage II (T3N0; n=227, 33 events). (C) Stage III (N1–2; n=233, 92 events). The training-cohort median linear predictor (1.011232) was applied unchanged within all three stages. Low- and high-risk groups were compared using log-rank tests.
