# Supplementary material for: Preoperative CT-derived liver fat fraction and visceral adipose tissue index enhance postoperative recurrence risk stratification in colorectal cancer
Source: Front Oncol. 2026 Sep 15;16:1944392. doi: 10.3389/fonc.2026.1944392 (PMC13619508; doi:10.3389/fonc.2026.1944392)
Supplement: Supplementary file 2 [file Table1.docx]

**Supplementary Tables S1-S5**

**Supplementary Table S1. Reproducibility of CT-derived VATI and LFF measurements**

| **Biomarker** | **Assessment** | **N** | **Measurement summary** | **ICC (95% CI)** | **Mean difference** | **95% limits of agreement** | **Wilcoxon P** | **Pearson r** | **Availability / interpretation** |
| --- | --- | --- | --- | --- | --- | --- | --- | --- | --- |
| VATI | Interobserver | 50 | Reader 1 vs Reader 2; same mid-L3 workflow | 0.995 (0.991–0.997) | Not available | Not available | Not available | Not available | Excellent cross-reader agreement; only the ICC was retained for this analysis. |
| VATI | Intraobserver | Not available | No archived same-reader repeat VATI segmentation | Not estimable | Not estimable | Not estimable | Not estimable | Not estimable | Could not be calculated retrospectively from the locked dataset. |
| LFF | Interobserver | 525 | Reader 1: 3.08 ± 4.11%; Reader 2: 3.17 ± 4.62% | 0.856 (0.822–0.883) | -0.092% | -4.689% to 4.505% | .463 | 0.862 | Good interobserver agreement; no significant paired difference. |
| LFF | Intraobserver | 525 | Reader 1 initial: 3.08 ± 4.11%; repeat: 3.09 ± 4.56% | 0.896 (0.870–0.917) | -0.013% | -3.895% to 3.868% | .888 | 0.901 | Good intraobserver agreement; no significant paired difference. |

**Notes.** ICCs are single-measure absolute-agreement intraclass correlation coefficients. LFF 95% CIs used 10,000 bootstrap resamples. Formal outcome blinding and reader specialty/experience were not documented. Same-reader repeat VATI segmentation was not archived, so VATI intraobserver ICC was not estimable. LFF Bland–Altman plots are provided in Supplementary Figure S1.

**Supplementary Table S2. Model selection, collinearity, penalized analysis, internal validation, and robustness diagnostics**

**A. Variance inflation factors across all 16 prespecified candidates**

| **Variable** | **R² from other predictors** | **VIF** |
| --- | --- | --- |
| Sex | 0.077637 | 1.084 |
| Age | 0.068217 | 1.073 |
| T stage | 0.131048 | 1.151 |
| N stage | 0.213155 | 1.271 |
| CEA | 0.177684 | 1.216 |
| CA19-9 | 0.167554 | 1.201 |
| PNI | 0.218935 | 1.28 |
| LVI | 0.220863 | 1.283 |
| Histologic grade | 0.121947 | 1.139 |
| BMI category | 0.037516 | 1.039 |
| Tumor location | 0.120007 | 1.136 |
| IMATI | 0.696789 | 3.298 |
| SATI | 0.688275 | 3.208 |
| VATI | 0.150872 | 1.178 |
| VSR | 0.636033 | 2.748 |
| LFF | 0.286512 | 1.402 |

**B. Ten-fold cross-validated LASSO Cox coefficients**

| **Variable** | **Coefficient at lambda.min** | **Selected at lambda.min** | **Coefficient at lambda.1SE** | **Selected at lambda.1SE** |
| --- | --- | --- | --- | --- |
| Sex | 0 | No | 0 | No |
| Age | 0 | No | 0 | No |
| T stage | 0.117751 | Yes | 0 | No |
| N stage | 0.434389 | Yes | 0.336652 | Yes |
| CEA | 0.204873 | Yes | 0.122265 | Yes |
| CA19-9 | 0.235505 | Yes | 0.184272 | Yes |
| PNI | 0.066285 | Yes | 0.014453 | Yes |
| LVI | 0.314365 | Yes | 0.207543 | Yes |
| Histologic grade | 3.1e-05 | Yes | 0 | No |
| BMI category | 0.014864 | Yes | 0 | No |
| Tumor location | 0.12578 | Yes | 0.055317 | Yes |
| IMATI | 0 | No | 0 | No |
| SATI | 0.010212 | Yes | 0 | No |
| VATI | 0.292723 | Yes | 0.156926 | Yes |
| VSR | -0.173034 | Yes | 0 | No |
| LFF | 0.730523 | Yes | 0.497529 | Yes |

**Notes.** Cross-validation used event-stratified folds. lambda.min=0.0199472 and lambda.1SE=0.06062343. All five primary predictors had non-zero coefficients at lambda.1SE.

**C. Bootstrap internal validation of the locked five-predictor model**

| **Metric** | **Estimate** | **95% CI / note** |
| --- | --- | --- |
| Bootstrap resamples | 1000 | Locked five-predictor Cox model; coefficients re-estimated in each resample |
| Reference apparent C-index | 0.851 | As reported in the manuscript |
| Mean bootstrap optimism in C-index | 0.01179 |  |
| Optimism-corrected C-index | 0.83921 | 0.798-0.879 |
| Optimism-corrected calibration slope | 0.92729 | 0.711-1.126 |
| Uniform coefficient shrinkage factor | 0.92729 | Derived from optimism-corrected calibration slope |

**D. Nine-variable candidate-model estimates**

| **Variable** | **Adjusted HR** | **95% CI** | **P value** | **Primary-model status** |
| --- | --- | --- | --- | --- |
| T3 vs T2 | 2.343 | 0.522–10.509 | 0.266 | Not retained |
| N1–2 vs N0 | 2.421 | 1.247–4.699 | 0.009 | Retained |
| CEA abnormal | 1.613 | 0.890–2.924 | 0.115 | Not retained |
| CA19-9 abnormal | 1.837 | 1.023–3.299 | 0.042 | Retained |
| PNI positive | 1.194 | 0.669–2.131 | 0.548 | Not retained |
| LVI positive | 2.226 | 1.221–4.057 | 0.009 | Retained |
| VATI, per 1 SD | 1.423 | 1.119–1.811 | 0.004 | Retained |
| LFF, per 1 SD | 2.112 | 1.612–2.766 | <0.001 | Retained |
| Rectum vs colon | 1.476 | 0.854–2.550 | 0.163 | Not retained |

**E. Schoenfeld-residual proportional-hazards tests**

| **Variable** | **Chi-square** | **df** | **P value** | **Interpretation** |
| --- | --- | --- | --- | --- |
| N stage | 0.013581 | 1 | 0.907225 | No evidence of PH violation |
| CA19-9 | 0.146351 | 1 | 0.702047 | No evidence of PH violation |
| LVI | 0.136849 | 1 | 0.711433 | No evidence of PH violation |
| VATI (per 1 SD) | 3.7e-05 | 1 | 0.995131 | No evidence of PH violation |
| LFF (per 1 SD) | 0.117703 | 1 | 0.731539 | No evidence of PH violation |
| GLOBAL | 0.455707 | 5 | 0.993655 | No evidence of overall PH violation |

**F. Exploratory biomarker-by-clinical-factor interactions in combined validation**

| **Biomarker** | **Clinical factor** | **Interaction HR** | **95% CI** | **Nominal P** | **BH q value** | **Interpretation** |
| --- | --- | --- | --- | --- | --- | --- |
| VATI | N stage | 0.798 | 0.427-1.494 | 0.481 | 0.642 | No interaction after multiplicity correction |
| VATI | CA19-9 | 0.519 | 0.277-0.973 | 0.041 | 0.163 | No interaction after multiplicity correction |
| VATI | LVI | 0.571 | 0.301-1.083 | 0.086 | 0.172 | No interaction after multiplicity correction |
| VATI | Tumor location | 1.195 | 0.516-2.768 | 0.677 | 0.774 | No interaction after multiplicity correction |
| LFF | N stage | 1.03 | 0.553-1.919 | 0.925 | 0.925 | No interaction after multiplicity correction |
| LFF | CA19-9 | 1.344 | 0.715-2.525 | 0.358 | 0.573 | No interaction after multiplicity correction |
| LFF | LVI | 0.54 | 0.274-1.064 | 0.075 | 0.172 | No interaction after multiplicity correction |
| LFF | Tumor location | 2.746 | 1.210-6.232 | 0.016 | 0.126 | No interaction after multiplicity correction |

**Notes.** No biomarker interaction remained significant after Benjamini–Hochberg correction (minimum q=0.126). In the training candidate model, CEA×CA19-9 was not significant (interaction HR 0.495, 95% CI 0.148–1.655; Wald P=0.253; likelihood-ratio P=0.264). T×N was not reliably estimable because the T2/N1–2 stratum contained 5 patients and no events. With 64 training events, EPV was 7.1 for the nine-variable candidate model and 12.8 for the final five-predictor model. All 16 candidate predictors, event status, and follow-up duration were complete in the 525-patient analytic dataset; no imputation was required.

**Supplementary Table S3. Baseline characteristics by RFS-event status within each cohort**

| **Cohort** | **Variable** | **No RFS event, n** | **No RFS event** | **RFS event, n** | **RFS event** | **P value** |
| --- | --- | --- | --- | --- | --- | --- |
| Internal training | Age, years | 196 | 68.00 [58.00-74.00] | 64 | 67.00 [55.00-75.00] | 0.385 |
| Internal training | Male sex | 196 | 91 (46.4) | 64 | 33 (51.6) | 0.475 |
| Internal training | T3 stage | 196 | 166 (84.7) | 64 | 62 (96.9) | 0.008 |
| Internal training | N1–2 stage | 196 | 70 (35.7) | 64 | 49 (76.6) | <0.001 |
| Internal training | Elevated CEA | 196 | 63 (32.1) | 64 | 39 (60.9) | <0.001 |
| Internal training | Elevated CA19-9 | 196 | 31 (15.8) | 64 | 29 (45.3) | <0.001 |
| Internal training | PNI positive | 196 | 67 (34.2) | 64 | 38 (59.4) | <0.001 |
| Internal training | LVI positive | 196 | 70 (35.7) | 64 | 41 (64.1) | <0.001 |
| Internal training | VATI | 196 | 36.63 [31.51-44.44] | 64 | 42.09 [32.54-47.10] | 0.016 |
| Internal training | LFF, % | 196 | 1.37 [-0.37-4.56] | 64 | 6.80 [3.40-8.38] | <0.001 |
| Internal training | Rectal cancer | 196 | 51 (26.0) | 64 | 32 (50.0) | <0.001 |
| External validation | Age, years | 156 | 66.00 [58.00-73.25] | 52 | 66.00 [58.00-72.50] | 0.844 |
| External validation | Male sex | 156 | 87 (55.8) | 52 | 24 (46.2) | 0.229 |
| External validation | T3 stage | 156 | 124 (79.5) | 52 | 51 (98.1) | <0.001 |
| External validation | N1–2 stage | 156 | 55 (35.3) | 52 | 38 (73.1) | <0.001 |
| External validation | Elevated CEA | 156 | 54 (34.6) | 52 | 36 (69.2) | <0.001 |
| External validation | Elevated CA19-9 | 156 | 27 (17.3) | 52 | 21 (40.4) | <0.001 |
| External validation | PNI positive | 156 | 63 (40.4) | 52 | 36 (69.2) | <0.001 |
| External validation | LVI positive | 156 | 50 (32.1) | 52 | 37 (71.2) | <0.001 |
| External validation | VATI | 156 | 37.84 [32.01-46.14] | 52 | 43.05 [33.28-47.10] | 0.164 |
| External validation | LFF, % | 156 | 2.24 [0.21-4.34] | 52 | 6.88 [3.40-7.70] | <0.001 |
| External validation | Rectal cancer | 156 | 43 (27.6) | 52 | 20 (38.5) | 0.139 |
| Prospective validation | Age, years | 44 | 61.00 [54.50-71.00] | 13 | 67.00 [59.00-71.00] | 0.253 |
| Prospective validation | Male sex | 44 | 26 (59.1) | 13 | 10 (76.9) | 0.333 |
| Prospective validation | T3 stage | 44 | 33 (75.0) | 13 | 11 (84.6) | 0.71 |
| Prospective validation | N1–2 stage | 44 | 16 (36.4) | 13 | 5 (38.5) | 0.89 |
| Prospective validation | Elevated CEA | 44 | 9 (20.5) | 13 | 6 (46.2) | 0.064 |
| Prospective validation | Elevated CA19-9 | 44 | 5 (11.4) | 13 | 10 (76.9) | <0.001 |
| Prospective validation | PNI positive | 44 | 16 (36.4) | 13 | 10 (76.9) | 0.013 |
| Prospective validation | LVI positive | 44 | 14 (31.8) | 13 | 10 (76.9) | 0.009 |
| Prospective validation | VATI | 44 | 36.19 [31.72-48.45] | 13 | 39.29 [30.85-41.12] | 0.902 |
| Prospective validation | LFF, % | 44 | 2.24 [0.14-4.27] | 13 | 8.23 [3.40-8.62] | <0.001 |
| Prospective validation | Rectal cancer | 44 | 19 (43.2) | 13 | 2 (15.4) | 0.103 |

**Notes.** Values are median [interquartile range] or n (%). P values compare patients with and without an RFS event within each cohort. CEA, carcinoembryonic antigen; CA19-9, carbohydrate antigen 19-9; LFF, liver fat fraction; LVI, lymphovascular invasion; PNI, perineural invasion; VATI, visceral adipose tissue index.

**Supplementary Table S4. Risk stratification, grouped calibration, and cutoff sensitivity analyses**

**A. Fixed training-median risk stratification**

| **Dataset/subgroup** | **N** | **Events** | **Low-risk N** | **Low-risk events** | **High-risk N** | **High-risk events** | **Log-rank P** | **High vs low HR (95% CI)** | **Cox P** |
| --- | --- | --- | --- | --- | --- | --- | --- | --- | --- |
| Internal training | 260 | 64 | 130 | 4 | 130 | 60 | <0.001 | 19.887 (7.209–54.855) | <0.001 |
| External validation | 208 | 52 | 105 | 5 | 103 | 47 | <0.001 | 12.550 (4.974–31.668) | <0.001 |
| Prospective validation | 57 | 13 | 29 | 1 | 28 | 12 | <0.001 | 14.289 (1.856–110.038) | 0.011 |
| All patients – colon cancer subgroup | 358 | 75 | 175 | 8 | 183 | 67 | <0.001 | 10.597 (5.069–22.156) | <0.001 |
| All patients – rectal cancer subgroup | 167 | 54 | 89 | 2 | 78 | 52 | <0.001 | 38.690 (9.415–158.988) | <0.001 |

**Notes.** The fixed cutoff was the training-cohort median linear predictor (1.011232) and was transported unchanged. Dichotomized analyses are secondary; continuous nomogram probabilities remain primary.

**B. Grouped calibration in the combined validation cohort**

| **Cohort** | **Risk group** | **Time, months** | **N** | **Events during follow-up** | **Mean predicted RFS-event probability** | **Observed KM RFS-event probability** | **Observed 95% CI lower** | **Observed 95% CI upper** |
| --- | --- | --- | --- | --- | --- | --- | --- | --- |
| Combined validation | Low | 12 | 134 | 6 | 0.018 | 0.032 | 0.012 | 0.083 |
| Combined validation | High | 12 | 131 | 59 | 0.159 | 0.196 | 0.137 | 0.276 |
| Combined validation | Low | 24 | 134 | 6 | 0.052 | 0.044 | 0.018 | 0.105 |
| Combined validation | High | 24 | 131 | 59 | 0.372 | 0.442 | 0.353 | 0.543 |

**Notes.** Observed probabilities are Kaplan–Meier estimates; corresponding plots are provided in Supplementary Figure S2.

**C. Alternative training-derived cutoffs**

| **Cutoff approach** | **Threshold** | **Cohort** | **Low-risk N** | **High-risk N** | **Low-risk events** | **High-risk events** | **High vs low HR (95% CI)** | **Log-rank χ²** | **Log-rank P** |
| --- | --- | --- | --- | --- | --- | --- | --- | --- | --- |
| Median linear predictor | 1.011232 | Training | 130 | 130 | 4 | 60 | 19.89 (7.21–54.86) | 66.63 | <0.001 |
| Median linear predictor | 1.011232 | Combined validation | 134 | 131 | 6 | 59 | 12.91 (5.56–29.97) | 59.38 | <0.001 |
| 25th percentile | 0.030709 | Training | 65 | 195 | 0 | 64 | Not estimable | 24.77 | <0.001 |
| 25th percentile | 0.030709 | Combined validation | 57 | 208 | 0 | 65 | Not estimable | 24.05 | <0.001 |
| 75th percentile | 2.085994 | Training | 195 | 65 | 19 | 45 | 9.92 (5.78–17.02) | 105.98 | <0.001 |
| 75th percentile | 2.085994 | Combined validation | 195 | 70 | 23 | 42 | 6.72 (4.02–11.23) | 71.14 | <0.001 |
| 12-month Youden | 2.125442 | Training | 197 | 63 | 20 | 44 | 10.57 (6.14–18.19) | 112.74 | <0.001 |
| 12-month Youden | 2.125442 | Combined validation | 200 | 65 | 24 | 41 | 6.75 (4.06–11.21) | 73.29 | <0.001 |
| 24-month Youden | 1.967391 | Training | 188 | 72 | 16 | 48 | 10.66 (6.03–18.86) | 103.03 | <0.001 |
| 24-month Youden | 1.967391 | Combined validation | 188 | 77 | 21 | 44 | 6.74 (3.98–11.40) | 67.98 | <0.001 |

**Notes.** All thresholds were derived in training and transported unchanged. The maximally selected log-rank threshold was 2.125442, identical to the 12-month Youden cutoff; it was outcome-optimized and was therefore not retained as a separate duplicated row. The 25th-percentile strategy produced no events in the low-risk group, so its Cox HR was not estimable.

**D. Biomarker cutoff derivation and external transport**

| **Biomarker** | **Horizon** | **Youden cutoff** | **Training AUC** | **Training sensitivity** | **Training specificity** | **External sensitivity** | **External specificity** |
| --- | --- | --- | --- | --- | --- | --- | --- |
| VATI | 12 months | 47.02 | 0.606 | 0.464 | 0.787 | 0.346 | 0.727 |
| VATI | 24 months | 41.12 | 0.605 | 0.620 | 0.636 | 0.643 | 0.535 |
| LFF | 12 months | 6.30 | 0.771 | 0.643 | 0.802 | 0.577 | 0.830 |
| LFF | 24 months | 6.01 | 0.758 | 0.680 | 0.839 | 0.548 | 0.828 |

**Notes.** Known-status ROC/Youden analysis excluded patients censored before the target horizon without an RFS event. At 12 and 24 months, the eligible samples were 230 (28 cases/202 controls) and 168 (50/118) in training, and 191 (26/165) and 141 (42/99) in external validation, respectively. Cutoffs were transported without re-optimization and are exploratory, horizon-specific biomarker thresholds rather than treatment thresholds.

**Supplementary Table S5. Candidate-model performance across cohorts**

Harrell C-index and time-dependent AUCs at 12 and 24 months are shown for the clinical-only, single-biomarker, combined clinical-imaging, and final nomogram models.

| **Model** | **Cohort** | **C-index** | **12-mo AUC** | **24-mo AUC** |
| --- | --- | --- | --- | --- |
| Clinical model | Internal training | 0.776 | 0.828 | 0.796 |
| Clinical model | External validation | 0.743 | 0.729 | 0.803 |
| Clinical model | Prospective validation | 0.748 | 0.707 | 0.775 |
| Clinical model | Combined validation | 0.746 | 0.730 | 0.793 |
| VATI model | Internal training | 0.577 | 0.611 | 0.603 |
| VATI model | External validation | 0.561 | 0.552 | 0.558 |
| VATI model | Prospective validation | 0.409 | 0.599 | 0.275 |
| VATI model | Combined validation | 0.533 | 0.561 | 0.509 |
| LFF model | Internal training | 0.743 | 0.764 | 0.761 |
| LFF model | External validation | 0.715 | 0.716 | 0.746 |
| LFF model | Prospective validation | 0.823 | 0.778 | 0.912 |
| LFF model | Combined validation | 0.730 | 0.720 | 0.771 |
| Clinical + VATI | Internal training | 0.788 | 0.849 | 0.810 |
| Clinical + VATI | External validation | 0.759 | 0.751 | 0.808 |
| Clinical + VATI | Prospective validation | 0.750 | 0.765 | 0.769 |
| Clinical + VATI | Combined validation | 0.758 | 0.753 | 0.796 |
| Clinical + LFF | Internal training | 0.833 | 0.886 | 0.857 |
| Clinical + LFF | External validation | 0.794 | 0.785 | 0.848 |
| Clinical + LFF | Prospective validation | 0.845 | 0.741 | 0.923 |
| Clinical + LFF | Combined validation | 0.802 | 0.780 | 0.853 |
| Final nomogram | Internal training | 0.851 | 0.918 | 0.878 |
| Final nomogram | External validation | 0.809 | 0.810 | 0.859 |
| Final nomogram | Prospective validation | 0.828 | 0.759 | 0.890 |
| Final nomogram | Combined validation | 0.813 | 0.807 | 0.860 |

**Notes.** AUC, area under the receiver operating characteristic curve; LFF, liver fat fraction; VATI, visceral adipose tissue index. The prospective cohort comprised 57 patients and 13 RFS events; its final-model C-index was 0.828 (95% CI, 0.675–0.947), its 12-month AUC was 0.759 (95% CI, 0.242–1.000), and its 24-month AUC was 0.890 (95% CI, 0.709–1.000), based on 20 evaluable patients. These estimates are exploratory. The clinical model comprised N stage, CA19-9, and LVI. The Clinical + VATI and Clinical + LFF models added the named imaging marker to these three clinical variables. The final nomogram comprised N stage, CA19-9, LVI, VATI, and LFF.
